# Precision Medicine in Inherited Retinal Disease: Advances, Challenges, and Future Directions

**DOI:** 10.3390/jpm16060292

**Published:** 2026-05-28

**Authors:** Thanansayan Dhivagaran, Fahad R. Butt, Krystal Grover, Krish Devgan, Kyran Sachdeva, Varounan Dhivagaran, Fatima Abid, Brendan K. Tao, Michael Balas, Ioannis Dimopoulos, Adil Bhatti

**Affiliations:** 1 Schulich School of Medicine & Dentistry, University of Western Ontario, London, ON N6A 5C1, Canada; 2 Faculty of Health Sciences, McMaster University, Hamilton, ON L8S 4L8, Canada; 3 Faculty of Medicine, University of Ottawa, Ottawa, ON K1H 8M5, Canada; 4 Faculty of Sciences, McMaster University, Hamilton, ON L8S 4K1, Canada; 5 Faculty of Science, Toronto Metropolitan University, Toronto, ON M5B 2K3, Canada; 6 Department of Ophthalmology & Vision Sciences, University of Toronto, Toronto, ON M4N 3M5, Canada; 7 Department of Ophthalmology, University of Ottawa, Ottawa, ON K1H 1C4, Canada

**Keywords:** precision medicine, inherited retinal diseases gene therapies, retina

## Abstract

**Background/Objectives:** Inherited retinal diseases (IRDs) represent a group of rare conditions characterized by significant clinical and genetic heterogeneity. Historically, the diagnosis of these conditions relied primarily on clinical presentation and imaging techniques. This literature review aims to discuss the current state of progress and ongoing challenges in applying precision medicine approaches to IRDs and to examine how advances in genetic testing have transformed diagnostics and opened new therapeutic avenues. **Methods**: This review examines the application of genetic testing methods to IRDs, with particular focus on next-generation sequencing (NGS) technologies. The review also evaluates current patient selection protocols that combine genetic confirmation, retinal structural evaluation, and detailed genetic counselling to achieve optimal therapeutic outcomes. **Results**: The implementation of NGS has significantly enhanced diagnostic capabilities for IRDs by enabling precise identification of specific genetic mutations. This advancement has paved the way for targeted therapeutic strategies, exemplified by Luxturna for RPE65-related IRDs. However, several barriers to broader adoption of precision medicine persist, including high costs, varied access to services, and complexities in interpreting genetic variants. **Conclusions**: While the continued development of innovative therapeutic modalities offers promise for expanding treatment options for IRDs, fully harnessing the potential of current and emerging therapeutic technologies requires addressing existing economic, technological, educational, and infrastructural challenges.

## 1. Introduction

Inherited retinal diseases (IRDs) are a group of rare, progressive conditions that can result in visual deficits or blindness due to dysfunction, degeneration, or abnormal development of the photoreceptors or the retinal pigment epithelium. IRDs encompass a wide range of disorders that differ in severity, age of onset, and rate of progression. They may affect central or peripheral vision and can present from early childhood to late adulthood. Although individually rare, IRDs are collectively important because of their chronic nature and long-term impact on vision and quality of life [1].

IRDs show significant clinical and genetic heterogeneity. For example, two patients with the same clinical diagnosis may experience different ages of onset, rates of progression, and severity of symptoms, while others with similar clinical presentations may have entirely different genetic mutations underlying their disease. These differences can come from principles such as allelic heterogeneity, where different mutations in the same gene can cause similar conditions, or locus heterogeneity, where mutations in different genes result in the same disease. This variability demonstrates why IRDs require a precision medicine approach [1].

In recent years, genetic testing has improved, changing the way clinicians approach IRDs. Rather than only relying on clinical features and imaging, there is now an increasing emphasis on identifying the genetic basis of disease in order to inform the diagnostic plan for the patient. This shift is one of the goals of precision medicine. Because IRDs are caused by a wide range of mutations across many genes, gene therapy aims to overcome this heterogeneity by targeting specific, disease-causing variants, either by replacing the defective gene, correcting the mutation, or modulating gene expression. This precision-based strategy enables more targeted and potentially effective treatments for patients [2].

The application of precision medicine for the treatment of IRDs is especially promising. However, understanding the results of genetic testing when variants of uncertain significance are involved is more challenging for doctors and patients. Exploring the techniques and methods used for interpreting IRDs and understanding the difficulty of linking genetic information with clinical outcomes is essential for advancements in precision medicine [2].

## 2. Background

The following section briefly outlines examples of IRDs to show this variability in clinical presentation and genetic mechanisms.

### 2.1. Retinitis Pigmentosa (RP)

Early signs of RP often include trouble seeing in low light (nyctalopia) and peripheral vision loss, which can later affect central vision as well. However, the age of onset and severity vary widely, and patients may also experience photophobia, near-sightedness, or colour vision defects. With over 80 genes implicated, RP can be inherited in autosomal dominant (20–25%), autosomal recessive (15–20%), or X-linked (10–15%) patterns, and rare cases of digenic inheritance have been reported. Even within the same gene, different mutations may have differing clinical presentations. For example, *ABCA4* and *BEST1* mutations can cause RP alongside other retinal dystrophies, and the same pathogenic variant can also present in a variety of ways across families [3].

### 2.2. Leber Congenital Amaurosis (LCA)

This IRD is a severe early-onset retinal dystrophy, which typically presents with visual impairment or blindness in infancy, though the severity of the disease and its symptoms, such as nystagmus, photophobia, or refractive errors, differ between patients. Additionally, fundus appearance and progression can differ, and extra-ocular features may occasionally be present in some patients. The condition is genetically diverse, with at least 20 genes involved, including *CEP290*, *CRB1*, *GUCY2D*, and *RPE65*. These genes affect different retinal pathways, such as phototransduction or ciliary function. Most cases are inherited in an autosomal recessive manner, though some rare forms follow an autosomal dominant pattern. This variation makes diagnosis complex, but also allows for new approaches to gene-specific therapy [4].

### 2.3. Stargardt Disease (STGD)

STGD is the most common inherited macular dystrophy, varying in the age of onset, severity, fundus appearance, and progression [5]. Some individuals present in childhood with rapid vision loss and impairments, while others are diagnosed in adulthood with slower decline [6]. Yellow-white pisciform flecks and macular atrophy may be absent early on, complicating diagnosis. Furthermore, full-field ERG and imaging findings differ across patients [6]. STGD is most commonly caused by mutations in *ABCA4*, but even among patients with similar mutations, phenotypes can vary [3].

Due to the high level of variability seen among different genotypes implicated in IRDs and how the condition clinically presents, precision medicine is a promising method of therapy for these unique conditions [3].

## 3. Genetics (WES/WGS, VUS)

Diagnosing inherited retinal diseases has become more reliant on genetic testing, as these diseases usually have mutations across a wide range of genes. With the growing availability of gene panels, whole-exome sequencing (WES), and whole-genome sequencing (WGS), clinicians now have more methods to uncover the molecular basis of retinal degeneration. Moreover, the cost of sequencing, particularly for WGS, has decreased dramatically in recent years, falling from over $4000 to approximately $200 per genome, thereby increasing its accessibility for clinical diagnostic use. However, these advances have challenges, especially when genetic findings do not clearly explain a patient’s symptoms. Variants of uncertain significance (VUS) further add to this complexity, making it hard to distinguish between benign variation and disease-causing mutations. At the same time, improved genetic resolution plays an important role in identifying patients eligible for new gene therapies, many of which are designed to target specific mutations or pathways involved in IRDs [7].

Gene panel sequences curated a list of genes known to be associated with IRDs. They are cost-effective and good for detecting known pathogenic variants, especially in populations with well-characterized mutation spectra. However, Panels can miss novel or rare variants outside the selected genes, which limits their use in highly heterogeneous populations or for discovering new disease genes [8].

WES sequences all protein-coding regions, capturing both known and novel variants across hundreds of IRD-associated genes [9]. WES is particularly valuable in genetically diverse populations or when initial gene panel testing is inconclusive [9]. It enables the discovery of new disease-causing genes and expands the mutation spectrum, which is seen in many IRD cohorts. However, WES may not detect deep intronic or regulatory variants and structural rearrangements [9].

WGS covers the entire genome, including coding, non-coding, and regulatory regions, and can identify variants which goes undetected by other methods [9]. WGS is being used more frequently for unresolved cases after panel or WES testing, although cost and data analysis still pose challenges [9].

Identifying clear links between different genetic variants and clinical features, known as genotype-phenotype correlations, is an important step in understanding inherited retinal diseases [10]. These correlations can allow researchers to understand how a disease might progress and help shape genetic counselling for families [10]. For example, individuals who carry two copies of a disease-causing mutation (homozygous variants) often present with more extreme symptoms or earlier onset when compared to individuals with only one copy (heterozygous) [10]. This pattern has been seen across multiple IRDs, including retinitis pigmentosa (RP), Leber congenital amaurosis (LCA), and Stargardt disease [10]. However, the relationship between genotype and phenotype is not always straightforward [10]. Certain genes, such as *PROM1*, *USH2A*, and *ABCA4*, have been associated with a wide range of clinical outcomes [9]. Sometimes, the same gene can lead to differing conditions, which are dependent on the specific mutation involved and whether it is inherited in a dominant or recessive manner [9]. Despite the growing availability of genetic testing, strong genotype-phenotype matches are still only found in a fraction of patients [9]. One large study conducted in an Indian population, for example, reported clear correlations in just 35% of cases, which shows the ongoing challenges in interpreting gene data and the influence of other factors, such as modifier genes or environmental exposures, on clinical presentation [9]. A major hurdle in the genetic diagnosis of IRDs is the high number of variants that are classified as variants of uncertain significance (VUS) [11]. These are genetic changes for which there is not enough evidence to determine whether they are actually involved in causing disease or are simply benign differences [11]. In one study, nearly 45% of all IRD-related variants were categorized this way, emphasizing how often clinicians are left with ambiguous results [11]. This uncertainty complicates not only how clinicians approach diagnosis but also how they support patients in making sense of their results, as it is challenging to offer confident answers about prognosis, recurrence risk, or eligibility for gene-specific therapies [11]. Sometimes, added steps like functional assays, segregation studies in relatives, or analysis of variant frequency in populations that are diverse can help clarify whether a VUS is likely to be harmful [11]. Still, these follow-up investigations are not always possible, especially in resource-limited environments or when family members are unavailable [11].

Beyond VUS, several other factors make genetic diagnosis of IRDs complex. Many IRDs share overlapping clinical features, making it hard to tell them apart just from symptoms alone. At the same time, extensive genetic heterogeneity means that mutations in dozens to hundreds of different genes can lead to similar retinal findings. The situation becomes harder to untangle when considering population genetics. For example, genetic differences might be more frequently observed in specific communities or in areas with high rates of consanguinity, which affects both how common the disease is and how it appears in individuals. Despite growing reliance on newer sequencing technologies, a portion of IRD cases remain without a clear genetic explanation. This points to missing genes still to be discovered, deep intronic or structural variants not captured by standard tests, or more complex modes of inheritance that are not fully understood as of right now, and need further research [8].

## 4. Therapies and Trials

Luxturna (voretigene neparvovec), developed by Spark Therapeutics, is the first gene therapy approved by the FDA for any inherited disease, specifically targeting vision loss caused by biallelic mutations in the *RPE65* gene [11]. This therapy is indicated for patients with Leber congenital amaurosis (LCA) and certain forms of retinitis pigmentosa (RP) due to *RPE65* mutations [11].

With regard to its mechanism of action, Luxturna uses an adeno-associated viral (AAV2) vector to insert the *RPE65* gene into retinal cells via subretinal injection, restoring the ability to produce the critical enzyme required for the visual cycle [11]. In clinical trials, Luxturna led to significant and durable improvements in functional vision, light sensitivity, and visual function, with benefits persisting for at least three years [11]. Many patients were able to get back some independence, such as navigating without canes and recognizing faces [11]. The FDA approved Luxturna in December 2017 following unanimous advisory committee support, representing a major turning point in the treatment of inherited retinal diseases [12]. The approval of Luxturna has accelerated progress for therapies for other IRDs. Several promising clinical trials are underway for genes implicated in RP, LCA, and related disorders:

*RPGR* (Retinitis Pigmentosa GTPase Regulator) mutations are a leading cause of X-linked retinitis pigmentosa. Multiple clinical trials are evaluating AAV-mediated gene replacement therapies for *RPGR*-associated RP [13]. Early results have shown some gains in visual function and safety, although long-term efficacy is still being assessed [14].

*CEP290* mutations are the most common cause of LCA. Efforts to treat *CEP290* include both gene augmentation and antisense oligonucleotide approaches [14]. Notably, antisense therapy (such as sepofarsen) aims to correct splicing defects, and clinical trials have shown some improvement in vision in treated patients [14].

*CRB1* mutations cause severe early-onset retinal dystrophies, including LCA and RP [14]. Preclinical studies and early-phase research trials are currently testing AAV-based gene replacement strategies for *CRB1*-related diseases [14].

Additional gene therapy trials are also happening for genes such as *CHM* (choroideremia), *USH2A* (Usher syndrome), *ABCA4* (Stargardt disease), and *RS1* (X-linked retinoschisis) [15]. Many of these therapies use AAV vectors, but alternative delivery systems and gene-editing approaches (such as CRISPR/Cas9) are also being looked into for genes with large coding sequences or dominant negative effects [15].

While challenges still remain for these therapies, as previously mentioned, precision medicine is a promising field of healthcare that could help better address the complications and complexities seen in IRDs and how individualized the condition is due to both its clinical and genetic heterogeneity seen among patients [15]. A summary of existing gene therapy preclinical and clinical trials is in Table 1.

## 5. Patient Selection

Clinically using gene therapy in IRDs requires a balance of eligibility and ethical considerations [33]. Precision medicine begins with patient selection protocols that are both biologically and ethically justified, all while managing patients and their families regarding the complexity, limitations and implications of therapy [33].

Eligibility for gene therapy requires, first, a genetic confirmation of disease etiology, primarily in the form of biallelic pathogenic variants in a causative gene [33]. As previously discussed, treatment with Luxturna requires two disease-causing mutations in the RPE65 gene, as established by FDA and EMA criteria [34]. Diagnoses often utilize NGS gene panels due to their efficiency and broad coverage across the genetic spectrum of IRDs [33]. The American Academy of Ophthalmology (AAO) recommends the inclusion of syndromic genes in all IRD panels, given the possibility of delayed systemic involvement, which further leverages the strength of NGS [33]. To reiterate, panels of patients with nonsyndromic presentations also include testing for syndromic genes, as systemic features such as hearing loss in Usher syndrome may emerge only later in life [33]. Furthermore, the accurate interpretation of results, especially in VUS, depends on the analytic sensitivity, quality of the reference database, and expert interpretation, underscoring the importance of collaboration among ophthalmologists, molecular laboratories, and certified genetic counsellors [35].

In addition to the presence of relevant genetic mutations, candidates may also need to demonstrate some level of preserved retinal architecture, as gene therapy cannot regenerate lost photoreceptors [36]. At this stage of testing, optical coherence tomography (OCT) is used to evaluate retinal structure, specifically assessing the continuity of the ellipsoid zone and central retinal thickness [36]. These measures are surrogates for photoreceptor viability and have been correlated with improved visual outcomes following subretinal gene delivery [37]. Full-field electroretinography (ERG) is also used to assess the function of rods and cones, supporting diagnosis and disease staging [37]. The quantitative readout provided by full-field ERG is crucial in confirming retinal involvement, especially in conditions such as LCA and RP [33]. This is an especially sensitive and vital test: even in patients with localized macular involvement, full-field ERG can detect subclinical, widespread degeneration that may affect outcomes and eligibility [33]. Furthermore, kinetic and static visual field testing can be used to help determine disease severity and baseline function, identifying patients who fall within regulatory or trial criteria, such as thresholds for legal blindness [33]. This can also serve as a measure of functional outcomes in trials [33]. Finally, fundus autofluorescence (FAF) and infrared reflectance imaging may support the identification of metabolically active RPE; however, FAF should be used with caution in macular dystrophies such as Stargardt disease, where short-wavelength exposure poses a theoretical risk of light toxicity [38].

Due to the progressive and irreversible loss of photoreceptors in IRD, treatment timing is critical, and regulatory approvals provide age-specific frameworks [38]. As explored earlier, Luxturna is currently intended for patients 12 months of age and older [39]. This is based on Phase 3 trial data and long-term follow-up studies, which indicate better visual outcomes in younger patients with less advanced disease [39]. Most ongoing trials in IRDs set age limits to account for the reliability of the test, disease progression and surgical risk [39]. For pediatric candidates, additional considerations include the feasibility of sedated imaging and ERG, as well as the challenges in assessing visual acuity and fields [39]. Clinical trials of different gene therapies may apply more stringent inclusion criteria [39]. The ongoing EDIT-101 trials for CEP290-related Leber congenital amaurosis (LCA10) include only individuals with a certain margin of residual retinal structure, as assessed by OCT [40]. Similarly, clinical trials targeting *RPGR*, *CRB1*, and *CHM* genes may also incorporate additional requirements, such as visual acuity thresholds, preservation of macular function, or exclusion of individuals who have undergone prior ocular surgeries [40].

Beyond initial selection, patients who are candidates for gene therapy require structured long-term follow-ups to assess safety, functional outcomes and disease progression [33]. Retinal imaging, ERF and visual field testing are periodically repeated to monitor the effect of treatment and detect late-onset complications [33]. These can include inflammation and subretinal fibrosis [33]. Clinicians, then, should prepare patients for immediate procedure and a lifelong trajectory of follow-up, re-evaluation and emotional adaptation [33].

This leads to a final, and perhaps most important, aspect of patient selection: comprehensive genetic counselling [33]. The importance of genetic counselling cannot be overstated as an essential and ethical part of patient selection [33]. In both trials and clinical use, patients express both hope and anxiety regarding gene therapy decisions [33]. As such, patients and families may assume curative outcomes. Instead, counselling helps reframe expectations towards stabilization or modest improvement [33]. For instance, in the Luxturna trials, although improved functional vision was observed in many patients, outcomes varied depending on age, disease stage, and baseline function [41]. Genetic counselling is especially vital in IRD, where VUS are frequently encountered and more than 80 genes can contribute to overlapping phenotypes [41]. Here, results require careful and nuanced interpretation and explanation to avoid inappropriate exclusion of false hope [41]. The AAO recommends that post-test counselling be conducted by clinicians or geneticists familiar with ocular genomics and ideally be reoffered throughout life as new therapies and variant classifications evolve [33]. Counselling must address psychosocial dynamics that patients and patient families often grapple with, such as feelings of hope, fear, guilt, and grief [33]. These may emerge both pre- and post-therapy, especially in families navigating decisions for children or those living with a progressive disability [33]. Support must not end with the disclosure of test results, and longitudinal counselling (and re-counselling) align with the best ethical practices to support informed and supported autonomy [33].

## 6. Barriers

Although gene therapy holds promising advancements for IRDs, several barriers must be overcome before it can be widely implemented [33]. These barriers span economic, infrastructure, technical and informational aspects [33].

### 6.1. Economic

Firstly, the high cost of gene therapy presents a significant barrier to access [42]. In the United States, Luxturna is priced at approximately $850,000 for both eyes, posing challenges at both the system and patient levels [42]. From the insurers’ perspective, health technology assessments are complicated by the high upfront cost coupled with uncertain outcomes [42]. These problematize payers’ coverage and affect access to gene therapy [43]. However, even when insurance is available, IRD patients often face restrictive costs, especially within the broader scope of the economic burden associated with IRDs [43]. Children with IRDs have 40% higher health expenses than their peers, owing mainly to increased outpatient eye care and psychiatric services [44]. The lifetime cost per person with IRD was estimated at $5.2 million in Australia, with 87% of the cost arising from societal costs, including loss of income for both patients and their caregivers [45]. Unfortunately, such financial burdens and patterns often persist into adulthood for those with IRDs as well [45]. While employment rates for adults with IRDs in Singapore are similar to those of the general population, the income for those with IRDs remains significantly lower [46]. In Canada, individuals with IRDs are 24% less likely to hold paid employment compared to the national average [47]. Furthermore, the estimated per-person societal costs associated with IRDs in Canada range from US$16,470 to over US$275,000 annually [47]. Lost productivity, diminished quality of life, and informal caregiving responsibilities contribute to up to 98% of these costs [48]. Thus, financial inaccessibility encompasses not only the high price of treatment but also the broader economic vulnerability experienced by individuals living with IRDs [48].

### 6.2. Infrastructural

Furthermore, the actual availability and distribution of centres and trained professionals vary globally and influence accessibility [49]. In Portugal, for example, genetic testing was found to be only available in 54.5% of centres, and of the 26 centres studied, only four actually used the national IRD registry [49]. Moreover, 83.4% of IRD patients experienced a turnaround time of 4 to 9 months for genetic test results [49]. Similarly, in Italy, patients with RPE65-related IRDs often consulted up to 10 healthcare professionals before finding an appropriate specialized centre [49]. This highlights that even when a centre is available, in the absence of a well-organized referral network, barriers to diagnosis and treatment persist [50]. Such a trend is also prevalent in Canada, where a study of 408 patients and caregivers found challenges in accessing genetic testing and specialized care, with many patients reporting delays in diagnosis and treatment [51]. While there are more specialized centres for IRD gene therapy in the United States, access remains uneven [51]. Centres such as the Bascom Palmer Eye Institute and the Massachusetts Eye and Ear Infirmary are concentrated in specific regions, which may lead to disparities in access for patients residing in rural or underserved areas [51].

### 6.3. Technical

Even once a centre is located, certain barriers persist [52]. The success of gene therapy in IRDs is dependent on the precise delivery and sustained expression of the therapeutic gene. As touched on briefly, variability in vector transduction efficiency, potential immune response and need for viable target cells can complicate treatment outcomes [52]. For example, the efficiency of AAV vectors can be affected by the presence of pre-existing neutralizing antibodies that can vary among individuals [52]. In some cases, the introduction of viral vectors can activate the immune response and cause inflammation, leading to a potential reduction in efficacy [52]. In the context of IRDs, these immune reactions can exacerbate retinal degeneration and present as gene therapy-associated uveitis (GTU), which has been reported in clinical trials [53]. In regard to the aspect of viable target cells, identifying patients early is crucial for the effective implementation of therapy. Leber congenital amaurosis and achromatopsia, for example, present preserved retinal structure for extended periods and are more ideal candidates for gene therapy when compared to an IRD such as retinitis pigmentosa, which presents with rapid retinal degeneration [54,55,56].

#### Informational

On a broader level, a lack of awareness and a clear understanding of gene therapy among patients and healthcare providers can further impede its implementation [57]. Misconceptions and limited access to accurate information may lead to barriers at a societal and mental level [57]. An Australian survey by Mack et al. probing the perspectives of individuals with IRDs found that only 28.3% of participants agreed they have good knowledge of gene therapy [57]. Though 86.9% were able to differentiate between an experimental treatment and an approved therapy, 52.9% incorrectly believed that gene therapy and stem cell therapy are the same. In a study with similar themes by Britten-Jones et al., only 37% of participants correctly responded that gene therapy for the eye is not suitable for all stages of the disease, and only 48% knew that the primary goal of the therapy is to slow the progression of the disease [58]. Such knowledge gaps are also observed in healthcare providers themselves [58]. A survey of optometrists in Australia and New Zealand found that only 38% of participants correctly identified the primary goal of gene therapy (slowing the progression of disease), and 81% indicated a lack of clarity on referral pathways as a key barrier [59].

Knowing this, efforts to address informational gaps are crucial for supporting decision-making and improving the implementation of gene therapy [58]. Britten-Jones et al. also found that individuals who self-reported good knowledge of genetic therapy were approximately three times more likely to feel confident in managing their own or their dependent’s eye care following testing [58]. One resource to enhance patient education is the Foundation Fighting Blindness annual VISION conference for patients and families [47]. The conference offers a place to connect with the community, learn about the latest research and treatments and explore new clinical trials and products within a supportive environment [47].

## 7. Future Directions

Gene therapy, as an emerging technology, still faces some degree of limitations—whether that be immunogenicity, restricted vector capacity or concerns about long-term efficacy—and so fosters a centre for further innovation. CRISPR-Cas9 gene editing, antisense oligonucleotides (ASOs), optogenetics and multi-omics approaches all hold potential, with each technology harbouring its own advantages and challenges, a few of which are discussed below [60].

### 7.1. CRISPR-Cas9

CRISPR-Cas9 is a two-component genome-editing system founded on a single-guide RNA (sgRNA) directing a Cas9 endonuclease to specific DNA sequences in order to generate a site-specific double-strand break (DSB) [60]. This break is then repaired by the cell and results in targeted insertions, deletions or precise replacements [60].

Applying CRISPR-Cas9 to IRDs has accelerated, originally beginning with using animal models with relevant mutations to aid in developing therapeutics that can edit photoreceptor or RPE cells in vivo [61]. A landmark preclinical study by Maeder et al. explored EDIT-101, an AAV-delivered CRISPR-Cas9 system aiming to edit the mutation responsible for LCA10 [61]. EDIT-101 achieved sustained editing rates, even exceeding the presumed therapeutic threshold and restoring regular transcript expression for photoreceptor structure and function [61].

Since then, a Phase ½ clinical trial (BRILLIANCE) has been evaluating the safety and tolerability of subretinally delivered EDIT-101 in LCA10 patients [22]. Early interim data thus far have indicated on-target editing in patient-derived retinal cells and no serious adverse events. Though final data is pending, these findings support the feasibility of CRISPR-Cas9 therapeutics in the field of IRDs [22].

At its ideal, CRISPR-Cas9 for IRDs can include the potential for permanent correction of recessive or dominant-negative mutations with a single intervention, standing to eliminate the need for repeat dosing [62]. Furthermore, the programmable nature of sgRNAs offers precise targeting of mutations across diverse IRD-implicated genes [62]. However, off-target cleavage remains a concern: unintended DSBS can cause changes in nontarget loci with variable downstream effects [62]. Preexisting or induced immune responses against the bacterial Cas9 protein could also reduce efficacy and lead to retinal inflammation [62]. Finally, efficient delivery to photoreceptors or RPE cells requires optimized vectors, and the current usage of AAV introduces constraints for the inclusion of larger Cas9 orthologs [63].

That being said, CRISPR-Cas9 editing in other genetic diseases, such as sickle cell disease, has demonstrated reliable on-target gene correction and clinical benefit [64]. For applicable translation to IRDs, general improvements in the CRISPR field can also be applied, such as engineering high-fidelity Cas9 variants, smaller Cas9 orthologs with AAV and the development of non-viral delivery methods [65].

### 7.2. ASOs

Antisense oligonucleotides, or ASOs, are short synthetic strands of nucleic acids that can hybridize with specific pre-mRNA sequences to modulate splicing [66]. In IRDs, ASOs aim to bind to mutated exons and induce exon skipping, allowing the synthesis of a truncated but partially functional protein [66]. Dulla et al. demonstrated that an intravitreal delivery of ASOs in a murine retinitis pigmentosa model successfully skipped the mutated exon [66]. This resulted in a 35–45% restoration of full-length transcription and partial preservation of photoreceptor function [66].

Several ASO candidates for *CEP290*-related LCA are in the early stages of clinical development [67]. AON-3, now formulated as QR-110 or sepofarsen, targets the c.2991+1655A>G *CEP29*-mutation and was found to restore normal *CEP290* mRNA in patient-derived retinal organoids [67]. Based on these findings, a first-in-human trial is evaluating safety and tolerability in *LCA10* patients [67]. Interim data describe dose-dependent editing in patient-derived retinal cells and no serious adverse events attributable to Cas9 editing [17].

ASOs offer high specificity, and they can be designed with single-nucleotide precision [17,67]. Unlike permanent gene editing, the effects of ASO are reversible; ASO gradually degrades over weeks, allowing for dosing adjustments if adverse effects arise [17,67]. On the flip side of the same coin, however, ASO therapy requires repeated intravitreal injections to maintain therapeutic levels, leading to increased procedural burden and risks [17,67]. It is also worth noting that exon skipping often yields retention of only partial function, and off-target splicing effects are also possible [17,67]. However, in IRDs, even partial preservation could maintain functional vision for years, and thus, ASOs present a compelling intermediate between gene supplementation and permanent editing [17,67].

### 7.3. Optogenetics

Optogenetics aims to repurpose surviving inner retinal neurons (typically bipolar or ganglion cells) into light detectors by using microbial opsin genes [29]. Sahel et al. report a successful case [29]. A patient presenting with retinitis pigmentosa received an intravitreal injection with an *AAV2* vector encoding a red-shifted channelrhodopsin, ChrimsonR, under a bipolar-cell-specific promoter [29]. When the patient then wore specifically designed amber-filtered goggles, he was able to locate and count objects, detect moving people, and recognize high-contrast shapes, regaining abilities he had lost prior to treatment [29]. These abilities persisted at six months without significant inflammation or adverse events [29].

Preclinical work in nonhuman primates has confirmed that bipolar-cell-specific expression of ChrimsonR via *AAV2* can yield stable mutation-independent photoreception [68]. A canine model of progressive rod-cone degeneration found that intravitreal delivery of AAV2-ChR2 restored light-driven pupillary reflexes and coordination to moving stimuli [68].

It is worth noting that while intravitreal injection is less invasive than subretinal surgery, visual acuity remains low, and bulky goggles are required to deliver sufficiently intense, wavelength-specific light [29,68]. Device calibration and patient training can take several weeks to achieve reliable performance [29,68]. However, optogenetics offers one of the few viable near-term options for patients lacking residual photoreceptor functions and could be combined with future cell-replacement or gene-correction approaches [29,68].

### 7.4. Multi-Omics

Multi-omics integrates multiple molecular layers (genome, transcriptome, proteome, metabolome and epigenome) to build a comprehensive molecular picture of disease [69]. In IRDs, multi-omics has been employed to elucidate complex genotype-phenotype relationships and identify novel biomarkers for diagnosis and stratification [69]. Liang et al. generated a single-nucleus multi-omics atlas of adult human retinas, combining transcriptomics and chromatin accessibility profiles from more than 250,000 nuclei [69]. Integrating this data revealed a core module of IRD-associated genes, including photoreceptor-specific transcription factors and metabolic enzymes, which had not been apparent from genetics alone [69]. Similarly, Lei et al. applied proteomic and metabolomic profiling to the vitreous samples of RP patients to identify elevated oxidative stress markers and dysregulated lipid pathways, suggesting potential targets for neuroprotection [70].

Advantages of multi-omics include the sensitive detection of subclinical disease stages, the refinement of patient subgroups for clinical trials, and the discovery of drug-target pathways [69,70]. However, this approach is resource-intensive, comprising sample collection, high-throughput sequencing, mass spectrometry, and computational integration [69,70]. From a patient’s perspective, multi-omics can yield personalized prognoses and inform the tailoring of future therapies [69,70]. However, broad implementation hinges on developing streamlined and cost-effective assays [69,70]. In practice, multi-omics is thus an emerging, realistic adjunct to genetic testing, with the power to accelerate the diagnosis of unsolved IRDs and prioritize patients for mutation-specific interventions [69,70].

Lastly, prior to implementing precision medicine in IRD therapeutic strategies, safety and procedural risks should be explored and addressed [71]. A recent systematic review and meta-analysis found that AAV ocular gene therapy had a cumulative serious adverse event incidence of 8%, with most adverse events attributable to the surgical procedure used to deliver the vector [71]. Ocular inflammation related to high-dose ocular gene therapies was found to be generally transient and resolved upon administration of corticosteroids [71]. Despite these findings, adverse event reporting in ocular gene therapy trials is inconsistent, suggesting that adverse events may occur at a higher rate than reported [71]. Future trials should investigate methods for safe dose selection and optimal delivery, such as intraoperative assistance with optical coherence tomography, to avoid off-target exposure, inflammation, and mechanical injury to the retina [71].

## 8. Conclusions

Over the past decade, precision medicine has transformed the IRD landscape: NGS panels and complementary imaging modalities now enable genetic diagnoses in the majority of patients, and multi-omics approaches and functional assays deepen our understanding of variant pathogenicity. Informed patient-selection protocols have already yielded tangible clinical benefits, and treatments are increasingly emerging and becoming available.

Despite these advancements, certain challenges persist. A marked subset of IRD cases remains genetically unresolved due to the presence of deep-intronic, structural, or non-coding variants that elude standard assays. Long-term safety and efficacy data for gene-based intervention are still maturing, and high costs, bioinformatic needs and regional disparities continue to limit broad, equitable access.

To bridge these gaps, the field must develop efficient and effective technology pipelines, in addition to forging international consortia to harmonize patient registries, trial eligibility, and post-market surveillance. Sustaining collaboration among geneticists, clinicians, health economists, patient advocates, and policymakers, the promise of precision medicine can be leveraged to translate into durable, accessible treatment; ultimately, to give the gift of sight to as many IRD patients as possible.

## Figures and Tables

**Table 1:** Clinical and Preclinical Trials for Gene Therapies in Inherited Retinal Diseases.

Trial/Program	Condition	Gene Target	Stage/Status	Organization/Scientists	Trial/Literature Reference
AAV-RPE65 preclinical proof-of-concept (Briard dog)	RPE65-associated Leber congenital amaurosis/inherited retinal degeneration	*RPE65*	Preclinical large-animal proof-of-concept; supported human translation	University of Pennsylvania/Cornell investigators	Acland et al., Nat Genet 2001 [16]
RPE65 Phase I gene transfer	RPE65-associated LCA/inherited retinal degeneration	*RPE65*	Completed Phase 1	NEI/University of Pennsylvania/CHOP and collaborators	NCT00481546; NCT00516477
Luxturna (voretigene neparvovec-rzyl; AAV2-hRPE65v2)	Biallelic RPE65 mutation-associated inherited retinal disease	*RPE65*	FDA approved Dec. 2017; pivotal Phase 3 completed	Spark Therapeutics	NCT00999609; Russell et al., Lancet 2017 [17]
Voretigene neparvovec long-term follow-up	Prior recipients of AAV2-hRPE65v2	*RPE65*	Long-term observational follow-up	Spark Therapeutics/Novartis	NCT03602820; NCT03597399
RPGRORF15 canine proof-of-concept	X-linked retinitis pigmentosa model	*RPGR*	Preclinical large-animal rescue study	University of Pennsylvania/collaborators	Beltran et al., PNAS 2012 [18]
Codon-optimized AAV8-RPGR	X-linked retinitis pigmentosa models	*RPGR*	Preclinical mouse-model optimization	University of Oxford/collaborators	Fischer et al., Mol Ther 2017 [19]
AAV5-RPGR/botaretigene sparoparvovec Phase 1/2	RPGR-associated X-linked retinitis pigmentosa	*RPGR*	Completed Phase 1/2; safety and efficacy signals reported	MeiraGTx/Janssen	NCT03252847; Michaelides et al., Am J Ophthalmol 2024 [20]
BIIB112/cotoretigene toliparvovec Phase 1/2	RPGR-associated X-linked retinitis pigmentosa	*RPGR*	Completed Phase 1/2	Nightstar/Biogen	NCT03116113
LUMEOS Phase 3	RPGR-associated X-linked retinitis pigmentosa	*RPGR*	Phase 3; did not meet primary endpoint reported in 2025	Janssen/MeiraGTx	NCT04671433
AGTC-501/laruparetigene zovaparvovec Phase 1/2 (SKYLINE)	RPGR-associated X-linked retinitis pigmentosa	*RPGR*	Phase 1/2 clinical data published	AGTC/Beacon Therapeutics	NCT03316560; Yang et al., Am J Ophthalmol 2025 [21]
VISTA pivotal trial of AGTC-501/laru-zova	RPGR-associated X-linked retinitis pigmentosa	*RPGR*	Randomized pivotal/registrational trial	Beacon Therapeutics	NCT04850118
LANDSCAPE bilateral-administration study	RPGR-associated X-linked retinitis pigmentosa	*RPGR*	Phase 2 open-label bilateral-treatment study	Beacon Therapeutics	NCT07174726
Sepofarsen/QR-110/AON-3 Phase 1b/2	CEP290-associated LCA10 due to c.2991+1655A>G (p.Cys998X)	CEP290	Completed Phase 1b/2 antisense oligonucleotide study	ProQR/Laboratoires Thea/Sepul Bio	NCT03140969; Russell et al., Nat Med 2022 [17]
ILLUMINATE sepofarsen Phase 2/3	CEP290-associated LCA10	*CEP290*	Completed Phase 2/3	ProQR	NCT03913143
HYPERION sepofarsen Phase 3	CEP290-associated LCA10	*CEP290*	Phase 3; first participant dosed reported in 2025	Sepul Bio/Laboratoires Thea	NCT06891443
EDIT-101 BRILLIANCE	LCA10 due to CEP290 c.2991+1655A>G/IVS26 mutation	*CEP290*	Completed Phase 1/2 in vivo CRISPR-Cas9 editing trial	Editas Medicine	NCT03872479; Pierce et al., N Engl J Med 2024 [22]
AAV2-REP1 first-in-human choroideremia gene therapy	Choroideremia	*CHM/REP1*	Completed Phase 1/2 dose-escalation	University of Oxford/Nightstar	NCT01461213; MacLaren et al., Lancet 2014 [23]
STAR: BIIB111/AAV2-REP1/timrepigene emparvovec	Choroideremia	*CHM/REP1*	Completed Phase 3; primary endpoint not met	Nightstar/Biogen	NCT03496012; Bergland et al., Nat Med 2023 [24]
BIIB111 bilateral-administration safety study	Choroideremia	*CHM/REP1*	Completed safety study	Nightstar/Biogen	NCT03507686
BIIB111 long-term follow-up	Prior BIIB111-treated choroideremia participants	*CHM/REP1*	Long-term safety and efficacy follow-up	Biogen	NCT03584165
4D-110 intravitreal CHM gene therapy	Choroideremia	*CHM/REP1*	Phase 1 dose-escalation	Four-dimensional Molecular Therapeutics/Roche	NCT04483440
STELLAR: QR-421a/ultevursen	Usher syndrome type 2A/nonsyndromic RP due to USH2A exon 13 mutations	*USH2A*	Completed Phase 1/2 RNA exon-skipping trial	ProQR	NCT03780257
SIRIUS: ultevursen	Advanced RP due to USH2A exon 13 mutations	*USH2A*	Phase 2/3; terminated/transitioned after program changes	ProQR	NCT05158296
CELESTE: ultevursen	Early-to-moderate RP due to USH2A exon 13 mutations	*USH2A*	Phase 2/3; terminated/transitioned after program changes	ProQR	NCT05176717
LUNA: ultevursen	RP due to USH2A exon 13 mutations	*USH2A*	Recruiting Phase 2b, randomized sham-controlled	Sepul Bio/Laboratoires Thea	NCT06627179
SAR422459/EIAV-ABCA4	ABCA4-associated Stargardt disease	*ABCA4*	Completed Phase 1/2a lentiviral gene therapy	Sanofi/Oxford BioMedica/OHSU/Quinze-Vingts	NCT01367444; Parker et al., Am J Ophthalmol 2022 [25]
ASTRA: SB-007 dual-AAV protein-splicing therapy	ABCA4-associated Stargardt disease	*ABCA4*	Ongoing Phase 1/2; dose-expansion initiated	SpliceBio	NCT06942572
CELESTE: AAVB-039 dual-AAV ABCA4 therapy	ABCA4-associated Stargardt disease	*ABCA4*	Phase 1/2, multicenter open-label dose-escalation	AAVantgarde Bio	NCT07161544
VG801 mRNA trans-splicing gene therapy	ABCA4 mutation-associated recessive retinal dystrophy/Stargardt disease	*ABCA4*	Phase 1/2 first-in-human dose-exploration	VeonGen/ViGeneron	NCT07002398
OCU410ST/AAV5-hRORA modifier gene therapy	Stargardt disease/ABCA4-associated retinopathy	*ABCA4* pathway modifier, not ABCA4 replacement	Phase 2/3 pivotal modifier-gene trial	Ocugen	NCT05956626
AAV8-RS1 ocular gene transfer	X-linked retinoschisis	*RS1*	Completed Phase 1/2a intravitreal trial; safety/tolerability focus	National Eye Institute	NCT02317887; Cukras et al., Mol Ther 2018 [26]
rAAV2tYF-CB-hRS1	X-linked retinoschisis	*RS1*	Completed Phase 1/2 intravitreal trial; no measurable treatment effect	AGTC/OHSU and collaborators	NCT02416622; Pennesi et al., Ophthalmol Retina 2022 [27]
AAV2/4-RS1	X-linked retinoschisis model	*RS1*	Preclinical Rs1-KO mouse rescue study	University of Florida/collaborators	Scruggs et al., Mol Ther Methods Clin Dev 2022 [28]
LIGHTHOUSE: ATSN-201	RS1-associated X-linked retinoschisis	*RS1*	Phase 1/2/3; pivotal Phase 3 cohort underway in 2026	Atsena Therapeutics	NCT05878860
PIONEER: GS030-DP/ChrimsonR optogenetic therapy	Advanced nonsyndromic retinitis pigmentosa	Mutation-independent optogenetics; retinal ganglion-cell ChrimsonR	Phase 1/2a; first reported partial functional recovery	GenSight Biologics/Institut de la Vision	NCT03326336; Sahel et al., Nat Med 2021 [29]
ChR2 optogenetic preclinical studies	Rodent/canine retinal degeneration models	Mutation-independent optogenetics; *ChR2*	Preclinical proof-of-concept	Multiple academic groups	Gaub et al., PNAS 2014 [30]
ChrimsonR nonhuman-primate proof-of-concept	Late-stage retinal degeneration platform development	Mutation-independent optogenetics; ChrimsonR/ChrimsonR-tdT	Preclinical nonhuman-primate safety and functional-expression study	GenSight/Sorbonne/Institut de la Vision collaborators	Gauvain et al., Commun Biol 2021 [31]
RESTORE/MCO-010 optogenetic therapy	Advanced retinitis pigmentosa	Mutation-independent optogenetics; bipolar-cell MCO	Phase 2b randomized sham-controlled study; positive durability reports	Nanoscope Therapeutics	NCT04945772; Lam et al., Nat Med/PMC 2025 [32]

## Data Availability

No new data were created or analyzed in this study. Data sharing is not applicable to this article.

## Abbreviations

The following abbreviations are used in this manuscript:

Abbreviation	Full Term
AAV	Adeno-associated virus
ABCA4	ATP Binding Cassette Subfamily A Member 4
AON	Antisense oligonucleotide
ASO	Antisense oligonucleotide
BEST1	Bestrophin 1
CEP290	Centrosomal Protein 290
CHM	Choroideremia
CRB1	Crumbs Cell Polarity Complex Component 1
CRISPR	Clustered Regularly Interspaced Short Palindromic Repeats
DNA	Deoxyribonucleic acid
ERG	Electroretinography
FAF	Fundus autofluorescence
FDA	Food and Drug Administration
GUCY2D	Guanylate Cyclase 2D
IRDs	Inherited retinal diseases
LCA	Leber congenital amaurosis
NGS	Next-generation sequencing
OCT	Optical coherence tomography
PROM1	Prominin 1
RNA	Ribonucleic acid
RP	Retinitis pigmentosa
RPE65	Retinal pigment epithelium-specific 65 kDa protein
RPGR	Retinitis Pigmentosa GTPase Regulator
RS1	Retinoschisin 1
STGD	Stargardt disease
USH2A	Usherin
VUS	Variants of uncertain significance
WES	Whole exome sequencing
WGS	Whole genome sequencing
